# Reflections on the ethics of participatory visual methods to engage communities in global health research

**DOI:** 10.1080/11287462.2017.1415722

**Published:** 2017-12-20

**Authors:** Gillian F. Black, Alun Davies, Dalia Iskander, Mary Chambers

**Affiliations:** ^a^ Sustainable Livelihoods Foundation (SLF), Wynberg Cape Town, South Africa; ^b^ Kenya Medical Research Institute – Wellcome Trust Research Programme, Kilifi, Kilifi County, Kenya; ^c^ Centre for Tropical Medicine and Global Health, University of Oxford, Oxford, UK; ^d^ Department of Anthropology, University of Durham, Durham, UK; ^e^ Wellcome Trust Major Overseas Programme, Oxford University Clinical Research Unit, Ho Chi Minh City, Vietnam

**Keywords:** Participatory visual methods, community engagement, ethics, biomedical research, global health

## Abstract

There is a growing body of literature describing conceptual frameworks for working with participatory visual methods (PVM). Through a global health lens, this paper examines some key themes within these frameworks. We reflect on our experiences of working with with an array of PVM to engage community members in Vietnam, Kenya, the Philippines and South Africa in biomedical research and public health. The participants that we have engaged in these processes live in under-resourced areas with high prevalence of communicable and non-communicable diseases. Our paper describes some of the challenges that we have encountered while using PVM to foster knowledge exchange, build relationships and facilitate change among individuals and families, community members, health workers, biomedical scientists and researchers. We consider multiple ethical situations that have arisen through our work and discuss the ways in which we have navigated and negotiated them. We offer our reflections and learning from facilitating these processes and in doing so we add novel contributions to ethical framework concepts.

## Introduction

The need for ethical community engagement in biomedical research is well established (Dickert & Sugarman, 2005; King et al., 2014; Marsh, Kamuya, Rowa, Gikonyo, & Molyneux, 2008). Participatory visual methods (PVM) have been used widely over the past two decades to engage communities in public health (Gubrium, Hill, & Flicker, 2014; Mitchell & Sommer, 2016; Wang, Burris, & Ping, 1996). The time has come to explore the potential of these visual methodologies for public engagement in biomedical research.

The term PVM describes an array of facilitated processes that support participants to produce their own images or dramatisations. These visual outputs or performances can be used by the people who produce them to tell personal stories or visually describe their perspectives and lived experiences of issues that are relevant and important to them. Community-led theatre has been a favoured method of visual engagement which has evolved in its application over many years (Boal, 1985; Uimonen, 2012). Increasingly popular PVM approaches include body mapping (Gastaldo, Magalhães, Carrasco, & Davy, 2012), photovoice (Wang & Burris, 1997), digital storytelling (DST) (Lambert, 2013), photo-elicitation (Harper, 2002), video diaries (Bates, 2013) and the production of participatory video (Milne, Mitchell, & de Lange, 2012; Shaw & Robertson, 1997). PVM are aligned with the principles of collaborative learning. They invite discussion and debate by enabling audiences to learn about the experiences of others, and to respond with their own stories and perspectives. Where, how and with whom the outputs of PVM are shared depends on the wishes and consent of the participants that create or produce them (Rose, 2016). Because the drawings, stories and films that are produced through PVM can be of a very personal nature, each step of a PVM process requires careful consideration of the ethical implications.

Bringing PVM into community engagement for global health has catalysed an important transition from one-way, top-down health and science communication (Gubrium, 2009). By presenting their plays, stories, videos or photographs to researchers and scientists, community members can raise their hopes, concerns, expectations and questions about health and medical research in personal and powerful ways while avoiding direct confrontation. Through engaging with these visual media, health practitioners and researchers - as well as family and fellow community members – can gain deeper understanding about the perspectives, experiences, opinions and ideas of patients and research participants (Harper, 2002; Wallerstein & Duran, 2010). Under the right conditions, these engagement processes can spark new conversations and be exciting and valuable for all who take part. However, bringing marginalised community members together with “experts” can also introduce uncomfortable power dynamics (Gaventa, 1993). An important element of any PVM process involves working with the participants to make decisions about if, where, when and how groups should be brought together for effective and constructive interaction.

It has been argued that participatory approaches encourage medical research participants to express themselves in ways that are not made possible by formal interviews or focus group discussions (Wallerstein & Duran, 2010). The *Health in the Backyard* (HIB) project in Vietnam (http://healthinthebackyard.org) was linked to a large cohort study measuring transmission of zoonotic diseases in over 1000 people and their animals (Rabaa et al., 2015). For many years, these communities were regularly interviewed about their farming practises. A survey conducted by a government vet using structured questionnaires indicated that farmers report to officials whenever they need to dispose of dead animals. HIB used DST as a visual method to engage with a subset of project participants. Their digital stories revealed behaviours which had not been brought up during the formal surveys or interviews. A number of individuals showed images of dead pigs and poultry that had been thrown into a waterway, and some participants described how they sold their sick pigs or chickens cheaply in the market. This PVM process thus provided new evidence to inform researchers about what really happens – at least in some cases – to dead and diseased animals in rural farms. But it also raised concerns with project staff about the consequences for participants who revealed they conducted these “illegal” behaviours. Being involved in the production of their short films opened up some important ethical implications for the story tellers and facilitators that are discussed in more detail below.

The four authors of this paper have gained experience of working with PVM in four very different contexts to engage community members in public health and biomedical research processes. Table 1 provides a summary of the author’s affiliations and the projects they have lead which are referred to in this paper. We draw upon multiple and diverse scenarios experienced through facilitating community engagement initiatives in South Africa, Kenya, the Philippines and Vietnam and use an expanded conceptual framework to share our observations and reflections with respect to ethical considerations of working with PVM in global health. We initially explore the implications of catalysing change and who is brought on board in facilitating PVM process. We then explore issues that arise when choosing specific methods, producing and editing content and disseminating and viewing visual outputs. We then consider intersecting matters of informed consent, confidentiality, anonymity and vulnerability. We offer our reflections and learning from facilitating these processes and in doing so we add some novel contributions to ethical framework concepts.

**Table 1.** Summary of authors' affiliations, projects, and participatory visual methods experience.

Author	Affiliation	Projects described in this paper	PVM used
Gillian Black	Sustainable Livelihoods Foundation (SLF) Cape Town, South Africa http://livelihoods.org.za	Heart of the Matter (HOTM; 2016) – http://livelihoods.org.za/projects/the-heart-of-the-matter	HOTM: Photovoice, collective video and collaborative video were used to foster knowledge exchange and co-learning between urban township residents and biomedical scientists about causes and effects of cardiovascular disease and how it can be addressed
		Community participation in action for the prevention of tuberculosis and HIV in South Africa (PEP-TB II; 2015) http://livelihoods.org.za/projects/pep-tb-ii	PEP-TB II: Participatory theatre, photovoice and DST were used in a layered approach to raise awareness about tuberculosis (TB) and HIV in the townships of Capricorn and Seawinds in Cape Town, and to convey to community members the lived realities of care workers in this context
		Engaging a vulnerable South African community in tuberculosis and HIV/AIDS prevention through a participatory approach (PEP-TB I; 2013) http://livelihoods.org.za/projects/usaid-i	PEP-TB I: Participatory theatre and DST were used to engage community members in multi-drug-resistant TB and the association between TB and HIV in the township of Delft in Cape Town, with a focus on understanding the experiences and perspectives of youth
Mary Chambers	Oxford University Clinical Research Unit (OUCRU) Vietnam	Place of Change (POC; 2014 - 2015) – health issues (malaria, STIs, HIV) in vulnerable communities. http://factandfictionfilms.com/engage/place-of-change	POC: DST (film, photos, images). Participants story-boarded and participated in filming to various degrees
		Health in the Backyard (HIB; 2014–2016) – perceptions of zoonotic risk in farming communities http://www.healthinthebackyard.org	HIB: Participant-led photo stories and photo-elicitation to facilitate community discussion
		Beyond the Hospital (BTH; 2016) – patient’s experiences after leaving hospital with moderate to severe disabilities resulting from brain infection	BTH: Participants guided an interviewer to take photos which were subsequently used on posters to inform discussions with health care workers and patients
Alun Davies	Kenya Medical Research Institute (KEMRI) Wellcome Trust Research Programme (KWTRP) University of Oxford	Evaluating engagement between health researchers and school students (2014–2015)	Participatory video enabled secondary school students to convey their personal experiences of engagement with health science research at KEMRI and to speak out about their challenges in pursuit of education
Dalia Iskander	University of Durham	Re-imaging malaria (2012–2014)	Photovoice was used to conduct a photo-ethnography of young people’s malaria-related health practice in the Philippines

### Catalysing change

A core objective of working with PVM is to facilitate transformation or change at some level (Burns, Howard, Lopez-Franco, Shahrokh, & Wheeler, 2013; Plush, 2012). Change can take many forms, from therapeutic effects for individual participants to shifts in national policy. Wang and Burris (1994) describe how, as a result of engaging in a photo novella approach, marginalised women in the Yunnan Province of China were able to inform three major policy changes.

Although working with PVM can bring about change that is perceived positively by participants and other community members in the short term, it can also bring about unintended and sometimes adverse consequences in the longer term. The photovoice project facilitated by Iskander in the Philippines focussed on exploring children’s perspectives of malaria among the Palawan population. All participating children, and some of their parents, reported that their malaria-related behaviours had changed as a result of the children’s engagement in the project. Crucially, going through the photovoice process altered the way in which children interacted with their families as many of them (and their parents) reported that, following the project, parents and relatives were more likely to ask their children for health advice and to take a lead from them in terms of carrying out healthy behaviours (Iskander, 2015). Children and parents both expressed that the role of children within the family had grown to include health promotion. While they saw this as being a positive outcome, it raises questions regarding the ethics of changing family dynamics in the longer term. Among the Palawan ethnic group, gender and relative age strongly influence how people interact and make decisions. In addition, Palawan society is highly relational in terms of decision-making in the home. The opinions of elders are especially well respected because, as one father explained, “adults have eaten more rice meals in their lives”. During the malaria photovoice process, participants of different genders and ages interacted with each other in new ways in order to take, engage with and respond to photographs. In any context, it is important to consider the potential of PVM like photovoice to alter the dynamics of family structure, and the long-term implications of catalysing these alternations. Aggett worked with teenage girls in Honduras in a visual methods process that changed the participants’ status within their families and wider society. As a result, there were jealousies, and some parents and teenagers that were not included in the project complained about these changes (Aggett, 2006).

### Who comes on board

PVM processes are lengthy and intensive and they can be emotionally demanding. They usually call on the focussed commitment of both facilitators and participants over weeks or months. Certain parts of the methodology require many hours of work over multiple consecutive days. With this in mind, the ways in which the participants and co-facilitators of a participatory visual (PV) process are identified and recruited needs to be carefully thought through well in advance of embarking on any community engagement project (Black & Tusiime, 2014).

The *Heart of the Matter* (HOTM) project in South Africa sought to engage members of an urban township community in biomedical cardiovascular disease research. Through this project, Black and her colleagues found that working with the same participants over multiple, theme-related projects can help to foster relationships, strengthen group capacity, build trust and grow the momentum of collective action within a community. However, this “way in” to a PVM process can also introduce bias by narrowing the range of community members who are given opportunities to share their experiences and convey their opinions through participation. The HOTM project provided an example of how challenges can arise when participants are involved in more than one project simultaneously through over-stretching their available time and energy and their ability to commit to workshops, meetings or events.

At OUCRU in Vietnam, there is a requirement to recruit study participants for medical research and health/science engagement projects either through government officials or hospital staff. In some cases, these officials may be well known and trusted by the community members, such as the local vets who recruited farmers into the HIB project. In other cases, community members are asked to take part in engagement projects by officials that they do not know. Chambers and her team held concerns that, due to this recruitment approach by government officials, participants may feel obliged to join PVM projects with little idea about what they are signing up for. In response to this, the engagement team created multiple opportunities for participants to pull out of projects. In a further effort to ensure willing and informed participation, the OUCRU team have followed a dynamic approach to obtaining consent, whereby participants are iteratively asked to consider their agreement to be involved in a PVM project as their understanding of the project deepens and their visual outputs are developed. The concept of dynamic consent and why it is important for community engagement in global health is discussed in more detail below.

Through the KWTRP participatory video project in Kenya, Davies supported high school children to produce short films conveying their experiences of engagement with health science research at KEMRI, and to speak out about their challenges in pursuit of education. The films that the learners made were shared with fellow students, teachers and researchers, and made available on the internet. Students did not volunteer themselves to be part of this project but were purposively selected to represent different school types, genders, and the extent of their participation in the student engagement programme. Group discussions indicated that the majority of students who took part in the project enjoyed the experience, though participant observation revealed that some were keen to complete the video process quickly in order to attend other extracurricular activities. The number of students that were selected for the KWTRP project was relatively small. The selection process made earnest attempts to include a wide range of views, however, some students who were not chosen but wanted to take part reported that they felt excluded.

Local co-facilitators can be a valuable asset to PVM projects, especially where vulnerable or at-risk groups are involved. Their insight and relatively close connection to the issues being explored can foster participants’ confidence in sharing personal experiences. In Vietnam, community-based health workers assisted with the recruitment of people living with HIV into the Place of Change (POC) project and were active in encouraging them tell and illustrate their stories. A more challenging experience was encountered when bringing local people onto the facilitation team in the Philippines malaria project. Iskander trained school teachers in photovoice so that they could co-facilitate workshop sessions. In training, the teachers were keen to be involved and excited by the potentially empowering features of the photographic approach for their students. However, while sessions were being held they often remarked that they were too tired to participate and needed a break, while many had meetings to attend or work they needed to do at these times. In addition, despite the training they received, teachers indicated that they did not feel they were adequately familiar with the photovoice technique to assist the children. They described the lead researcher as the “real expert” and tended to go to her for instruction and guidance. The teachers also found it difficult to shift from their roles as authority figures and disciplinarians, and sometimes reacted with admonishment to student’s suggestions – especially if those suggestions differed from their own knowledge, or information that they were teaching in class. Reprimand and ridicule inhibited the children from expressing themselves fully and freely. These factors undermined the intended participatory and “community-led” nature of the project and highlight the need to critically evaluate the implications of involving local facilitators in PVM projects.

### Which method?

The most appropriate type of visual method to work with in a global health engagement activity is strongly influenced by the context of the project and the profile of those taking part. As far as possible, decisions about which PVM to use in a community engagement initiative should be made by the participants themselves. However, practical demands often mean that in reality, choices over the methods used are more likely to be the result of negotiations and compromises between participants and the process facilitators.

In the Philippines, the visual method that was chosen (photovoice) reflected the interests and skills of the research facilitator and the availability of equipment. The choice of method was also influenced by the requirements of numerous ethical review bodies and restrictions imposed by the project funders. In the end, the Palawan children who participated in the Exposing Multiple Malarias project reported that they enjoyed leaning about and using the photovoice approach. During the consenting process, community leaders and individuals remarked that the project would “leave something behind” for the community in terms of increased knowledge, skills and equipment related not only to malaria but also photovoice as a method of expression.

The community engagement team on the KWTRP project have a strong interest and five years’ experience in the use of participatory video for engagement with student participants, including the evaluation of engagement activities. Based on this experience, Davies and his colleagues surmised that participatory video would be an enjoyable means for students to share their views on engagement in medical research at KEMRI. PV has been lauded for its capacity to empower participants, and empowerment is recognised as an important ethical goal of community engagement (CTSA Community Engagement Key Function Committee, 2011). The facilitation team in Kenya thus considered that PV had the potential to gather evaluative data whist simultaneously emboldening the learner participants (Nemes, High, Shafer, & Goldsmith, 2007).

The strength of relationships between members of a group embarking on a PVM process should also be considered before choosing a method as this influences how much support group members are able to give each other. In 2015, SLF facilitated a community engagement initiative which aimed to understand the work experiences of community care workers (CCWs) in providing door-to-door treatment adherence support and monitoring for people living with TB and HIV. The aim of the CCW project was to illustrate and convey to community members and health professionals the daily realities of these grounded health providers. The facilitation team at SLF had considerable experience of working with a specific DST methodology and had pre-selected this approach for the CCW project. The chosen methodology is intensive and reflective, tending to result in the surfacing of powerful, deeply personal stories. Due to timeframe constraints, the duration of the care workers project was tight with limited time at the outset to explain the emotional implications of participation. Given the methodological approach taken in this case, on reflection, Black and colleagues recognised that certain members of this group would have benefitted from long-term professional counselling support, especially since the CCWs did not all know each other when the project began, and had no previous connection with SLF.

### The topic and content of PVM outputs

The position of a PVM process on the spectrum of participation strongly influences the content of the resulting visual outputs (Pauwels, 2011). How comfortable participants feel during the development of their media also has a major bearing on how willing they are to engage in sharing their personal or collective experiences and opinions with others.

The students that participated in the participatory video project in Kenya were given an open brief to make films and produce plays relating to their experiences at KEMRI and the challenges they faced in pursuing education. The learners’ films conveyed a wide range of student perspectives regarding their understanding of scientific research, and illustrated the difficulties they faced in pursuing education due to a lack of school fees and their parents’ hesitation about the value of education. Students also revealed several other significant challenges they faced in their day-to-day lives including unwanted sexual advances experienced on the way to or from school, unanticipated pregnancies and early marriages. The personal and intimate content of these visual outputs, enabled by the open brief, indicates that students were ready and keen to engage, and that they held a strong sense of ownership over the direction of the process. The opportunity to control the content of the films through storyboarding and editing, and to make final decisions about which films to share with wider audiences, fostered confidence among students to openly share their views. As the students developed the content for their videos nobody else was present except the KWTRP community engagement team who maintained a support facilitation role only. This freedom of expression was an important factor in the integrity of the video content. Participation in the editing process over time encouraged deeper discussion of the issues that students were facing which aided their reflections regarding what they wanted to include, delete or amend for the final versions. This reflective process also allowed time for students and facilitators to strengthen their relationship and create a conducive rapport for discussion and sharing of experiences.

The physical presence of people other than participants and facilitators during a PVM process can influence the content of the visual outputs. In the HIB DST project in Vietnam, the local government vets who partnered with the OUCRU team took pride in how much the farmers knew about animal diseases. They were concerned to “correct” the story boards or scripts that the participating farmers developed. Because the main aim of the HIB project was to gain a clearer understanding of practices and perceptions around risk-to-health behaviour in the community, this interference compromised the learning from the project, and was detrimental to the outcome. In many cases, the participants noticeably changed what they were saying. For example, one farmer informally told the process facilitators that he threw dead animals into a nearby canal, but he did not mention this when choosing photos and editing his photo story because a government vet was standing behind him at the time. Although some of the vets could understand the aims of the project and made efforts not to intervene, others had to be “invited for coffee” to prevent them pressurising the participants.

One of the aims of working with PVM is to create and exchange fresh learning that will stimulate new discussion and debate among participant groups and wider audiences. Black and her colleagues in Cape Town facilitated a participatory theatre project with a group of 20 township residents who were representative of their wider community and wanted to increase local knowledge about tuberculosis (Table 1; PEP-TB I). The first steps of the “script” development process revealed that the focus group already had a very good understanding about the symptoms and treatment of drug-sensitive TB, and that they wanted to learn more about the causes of drug-resistant TB and the association between TB and HIV. The play that they produced and went on to perform was based on these new learning areas and helped to generate new perspectives about TB and HIV among themselves and the people they performed to. Through this work, the facilitation team learned the value of eliciting and building upon pre-existing community knowledge for progressive health engagement.

The type of equipment used during a visual methods process can make a big difference to the content of PVM outputs (Wagner, 2011). If technology that is introduced to support the process is too complex, and requires the intervention of facilitators to operate it, this inhibits participants from directly creating their own visual content. In their facilitation of DST processes, the SLF community engagement team in South Africa have learned that participants are able to enjoy a much more hands-on and involved experience in the creation of their stories if they work with tablet technology as opposed to lap top computers (Black & Tusiime, 2014). However, in some cases, it may be necessary for the facilitation team to play a role in capturing images. For example, the BTH project in Vietnam engaged with patients who had left hospital but endured ongoing disabilities after treatment for brain infections. The community engagement team had anticipated that the participants would create their own photo diaries, however, it was quickly apparent that the patients were too weak and their carers too busy to hold the cameras. In compromise, the facilitator took photos as the carer or patient directed them.

### Dissemination and viewing of visual material

Audiences of media that are produced for health and science engagement through PVM processes might include the participants only, or may extend to their families and peers, other community members, researchers, local stakeholders such as health care providers and vets (as in the case of Vietnam), or policy- and decision-makers. In some cases, the choice of audience might be pre-defined by the intended purpose of a PVM project and the pre-planned use of its media outputs.

However, at the beginning of a PVM process, participants do not know what the final content of their media will include, so they cannot always pre-empt who they do or do not want to share them with. Even when audiences are pre-selected or chosen by PVM participants, the way that those audiences will respond is unpredictable (Mitchell, 2011). Health scientists quietly watched the films produced through the PV project in Kenya and afterwards reported that they were useful because they highlighted knowledge gaps and weaknesses in the schools engagement programme which could be improved upon. On the other hand, students who were not part of the video-making process that were invited to watch the films tended to become overwhelmingly excited at seeing their fellow class-mates on screen. This type of uncontrolled response made it difficult for the community engagement team to interpret or document audience reaction meaningfully.

### Informed consent and assent

Giving or gaining consent is important for maintaining rapport and relationships of trust between participants and researchers or facilitators in PVM processes and it is essential to ensuring the success of ongoing or subsequent research or engagement (Prosser, 2000; Rose, 2016 (Molyneux & Bull, 2013)). Whether or not participants can always fully understand what it is they are consenting to, is debatable (Wiles, Crow, Charles, & Heath, 2007). As discussed above, participants of a PVM process cannot pre-empt the full content of the media they will create before they finish it. Therefore it is appropriate for intermediaries to provide opportunities for participants to reconsider consent at multiple stages, especially at points where decisions need to be made about how, where and with who their visual outputs will be shared. This multi-stage process of permission review is referred to as dynamic consent and was first used in the field of bio-banking and human genetics research (Stein & Terry, 2013).

The participatory video project facilitated by KWTRP involved a complex and multi-layered process of dynamic consent. It required the sequential co-operation of multiple stakeholders including the parents of selected students, the students themselves, the principals of all participating schools and the sub-county education officer. Those learners who wanted to take part in the participatory video process, and their parents, were required to sign a consent form prior to attending the initial training workshop, with an understanding that students could opt out of the study at any time. At the end of the eight-week film-making process, students arrived at consensus regarding which of their films they wanted to show to broader audiences and make available on the internet. Their agreement to release those films was confirmed through a further layer of consent documentation. School principals then reviewed all the films and subsequently provided additional signed permissions to endorse the schools approval for the films to be released. Finally, the sub-county education officer reviewed the films to ensure that the content was suitable and appropriate for dissemination, at his discretion. This dynamic consent approach attempted to minimise risks for participants by providing them with ample opportunities for discussion and reflection – and the chance to change their minds – before arriving at final decisions regarding which of their films could be openly shared.

In the Vietnamese case studies, practitioners have also recognised the value and importance of dynamic consent. The OUCRU community engagement team found that some participant groups in the HIB project were initially reticent about their films being shown to a wider audience, but after completing their photostories and watching them within the project group, they were pleased with the finished products and changed their consent to allow a wider use of the films. On the other hand, a group of ethnic minority farmers who made videos about their experiences with malaria as part of the POC project narrowed their consent for which audiences could see their films. When they first made the films they gave consent for unrestricted usage of the media. However, 12 months later when the OUCRU community engagement team wanted to hold community screening sessions demonstrating how the films had been incorporated into an educational website, the farmers stipulated that the screenings should only be held in villages over 20 km away, to ensure that they were not recognised.

During the HOTM project in South Africa, participants were asked to take photographs in their home community that illustrated their daily food choices, and to write narratives describing their perspectives on the links between diet and heart health. Many participants captured close-range shots of people who could be easily identified in the photographs, and were described in the narratives as having diabetes or high blood pressure, or being victims of strokes or heart attacks. The output of this photovoice project was a full colour book. Hard copies of the book were disseminated widely in Cape Town and it was uploaded to the SLF and donor websites. It was therefore absolutely vital that every person who had been photographed was given the opportunity to see their photograph and its accompanying narrative before it was edited into the book. The SLF team involved the participant photographers in making sure that the subjects of their photographs were fully aware of the ways in which the book was to be distributed. The photographers were also asked to obtain written informed consent from the subjects they photographed. Thus the participants were required to act as ethical mediators as well as data producers. The project facilitators endeavoured to strengthen the integrity of this approach by ensuring that the photographers received prior training in consent procedures, were involved in drawing up the consent form, and that the consent forms were available in all local languages.

### Confidentiality, anonymity and vulnerability

Anonymity and confidentiality are key factors for facilitators and participants to consider in dynamic consent processes for PVM, as being identifiable in the resulting visual media may have repercussions for individuals and/or communities (Wiles, Clark, & Prosser, 2011). As far as possible the implications of identification should be discussed with the participant group early on in the process as their choices in this regard may have implications for the choice of PVM that is pursued. Of course, not all possible repercussions or implications can be predicted at the outset.

Working with drama or role-play methods can provide opportunities for participants to depersonalise and share sensitive views and experiences with broader audiences through the guise of fictional characters (Sloman, 2012). By taking this approach to engagement, participants may make themselves less vulnerable. In Vietnam, the OUCRU community engagement team used forum theatre with health care workers (HCWs) to help them explore some of the stressful issues connected to working with severely ill patients, in particular communicating with relatives. The BTH photo stories and interviews from patients who had recently been discharged from hospital provided a platform for the development of a loose “script”. The role-play method enabled HCWs to try out different ways of responding to stressed or angry relatives, allowing them to indirectly confront this challenging but prominent aspect of their work.

The Vietnamese POC project involved the participation of people living with HIV and women with STIs. Some of the participants were very concerned about confidentiality. The facilitators demonstrated how their films could contain open access images taken from the internet and photos of their shadows or backs rather than showing their faces in their films.

If they are handled with care, PVM processes can work against stigmatisation, destabilise the power relations implicit in traditional research methodologies and generate empathetic and reciprocal relations between health workers and historically marginalised populations (Ritterbusch, 2016). However, the preference that some participants have to remain anonymous in their PVM outputs is often driven by a sense of fear and vulnerability. Some groups or individuals may be particularly vulnerable, and the process of telling their story can make them even more so, or reinforce damaging stereotypes that are held against them. The community engagement teams in Vietnam and South Africa have learned about the benefit of inviting a trained counsellor to attend sessions or workshops and have found that this type of intervention works best if the counsellor is already known to the participants and remains in attendance throughout the whole process.

Self-awareness of vulnerability was evident among participants in the POC project in Vietnam in that some participants told “made up” life stories. The facilitation team were careful to affirm these stories as valid because they were the ones that the participants chose to tell.

In the photovoice project in the Philippines, school children took photographs of – and discussed – a range of their health-related practices. In their eagerness to engage in the project, the learners were not just passively recording their potentially risky health practices, but were purposively carrying them out in order to take pictures of them. For example, in all five participants groups, children took pictures of themselves or others around them being bitten by mosquitoes, allowing the insects to rest on their skin and even bite them in order to capture the moment in front of the camera. While the risk of malaria in the study context is relatively low, this example highlights ways in which PVM projects can compound the vulnerability of participants by increasing their exposure to risk.

The KWTRP project in Kenya demonstrated how the increased vulnerability of children, brought about by their participation in a PVM, process may not be recognised by facilitators or participants but instead noticed and acted upon by concerned people who are external to the project process. Whilst reviewing student-produced videos, a school principal shared his opinion that showing footage of a student expressing a local cultural belief about scientific research might raise concerns among community members who did not share this view. The principle feared that the student may be perceived as having a limited understanding of research. Following further discussion with the student, and with the student’s agreement, this clip was removed from the final film. This scenario highlights the importance and value of the participatory process itself as a means of understanding and appreciating diverse community views and sensitivities, as opposed to a sole reliance on the final media as a source of knowledge. When conducting DST with vulnerable groups such as people living with HIV or STIs, the OUCRU team invited trusted community-based health workers to be involved in the process. Not only were they able to support participants through the project, they were also able to follow up with individuals, potentially left emotionally raw after exploring and illustrating some very personal issues.

The vulnerability of community members may be eased in engagement processes that bring them together with scientists or health professionals if everyone involved is asked to open up and share information about personal practices or challenges. During the inception phase of the HOTM project in South Africa, cardiovascular disease research scientists and adult members of a poor township community were introduced to each other during a workshop held in the township setting. The facilitators asked all participants from both groups to create posters to illustrate and briefly describe their day-to-day diets. All participants were then invited to share their posters back to the whole group. The SLF team hoped that this approach would create a sense of parity and enable the community members to connect with the scientists by sharing the same type of personal information on the same platform, at the same time, in what the facilitators attempted to make a non-judgemental space. The majority of the township participants presented their posters to the whole group, talking openly and confidently about their eating habits and some of the things that they wanted to try and change. On the other hand, only one scientist came forward to share a poster with the rest of the group, and appeared very nervous to do so. It was clear from the content of their posters that the scientists were not trying to hide the fact that they did not have especially healthy diets. Their hesitation and embarrassment was more to do with the acute awareness that they had more choice, and more money to spend on luxury and convienience food items. In this case, it was the scientists who were made to feel ashamed and vulnerable. Through this experience, Black and her colleagues have learned that everyone who becomes closely involved in a PVM process can be exposed to vulnerability that is notably heightened by the visual nature of participation.

## Discussion

Our collective experience shows that sharing and discussing the outputs of PVM processes can foster co-learning, enabling researchers, health workers, research participants and researched communities to see and understand each other – and the health issues they face – in new ways. The projects we have undertaken in Kenya, the Philippines, South Africa and Vietnam illustrate the exciting diversity of engagement platforms that different PVM processes can provide. However, our work also highlights many intersecting ethical dimensions that need to be considered throughout the facilitation of PVM processes for community engagement in global health research.

The re-imaging malaria project in the Philippines has demonstrated the potential of visual methods to change culturally embedded family dynamics. Iskander's experience provokes important thinking about the implications of promoting children to become key consultants for health practice in traditional communities. It also resonates with some of the tensions and challenges that have been previously described with respect to doing creative visual research and engagement with children (Lomax, 2012; Drew, Duncan, & Sawyer, 2010).

All of the country case studies have shown how vital it is to consider the mechanisms that bring participants and facilitators into PVM processes, and the ways in which recruitment or selection can influence the experience of participation (Khanlou & Peter, 2005). In each country setting, we have noted how important it is for potential participants and facilitators to be aware of how much time they will need to invest in a PVM process before agreeing to become involved. The Kenyan project demonstrates how widening the scope of participation can be paradoxically and unintentionally exclusionary.

Our reflections illustrate some of the factors that influenced our selection of specific visual methods and describe how, in each case, this choice was made in advance by the facilitation team rather than the project participants. We recognise that this approach compromised the absolute level of participation in the various projects, but would argue that it enhanced the quality of the outputs due to the confidence of the PVM trainers and their ability to transfer technical skills. However, our experiences also serve to remind us that methods which elicit personal stories tend to dig deep emotionally, highlighting the importance of putting appropriate support structures in place for participants, should they become necessary. But the need for sensitivity and reflexivity does not stop with community participants. The challenges of navigating the vulnerabilities of medical researchers who become closely involved in community engagement projects have been illustrated through the HOTM project in South Africa. To avoid discomfort it is crucial that adequate investment is put into building trusting relationships within the participant group, and between the participants and the facilitators, and that conscious efforts are made to create safe spaces in workshop environments.

The experiences gained in the Philippines and Vietnam have shed light on the pressures that are introduced through the presence of authority figures during a PVM process and the ways in which they can influence the content of its outputs. We have also seen how the content of PVM outputs can be shaped by pre-determined audiences and that responses to this visual material can vary greatly depending on the profile of the audience. Pressures that exist at the outset of an engagement project and prescribed goals of donors or the facilitation team can steer the direction of a PVM process and influence the content of images, stories or dramatisation. If there are donor-related or institutional requirements that influence the ways in which visual outputs can be used by participants for their own purposes, the community engagement facilitators need to bring these factors to light and discuss them with participants as a priority as early as possible.

Collectively we have found that the purpose of engagement, the willingness of participants to be involved and the use of their visual outputs need to be revisited multiple times. Participants can only make an informed decision about whether they want to be recognised as a story teller, photographer or video maker when they have seen the final product for themselves, understand the ways in which the material might be shared and have had a chance think through the possible implications (Kombo, Sariola, Gichuru, Molyneux, Sanders & van der Elst, 2017). Within our four country cases, some participants really wanted their faces to be recognisable and their names to be clearly seen or heard in their stories or films. Others preferred to remain anonymous. Both of these preferences, and those that fall in between, need to be acknowledged and accommodated as visual outputs are being created.

The development sector has long emphasised the importance of fostering sustainability through training and capacity building. Sustainability is a core ethical issue for PVM processes as they mostly require access to technological equipment and accessories, and the necessary skills to use it (Wallerstein & Duran, 2010). The equipment needed to conduct a photovoice process was left with the participant teachers and students at the end of the malaria project in the Philippines, with the intention of enabling them – or the wider community – to experiment with the method in the future. However, to date teachers have reported that they have been unable to do so, citing reasons such as a lack of time but also basic resources such as batteries, and a lack of confidence in their own expertise. PVM also raise questions about the longevity of visual outputs regarding how they can be used for the benefit of participant communities after a specific project has ended. In an effort to address this issue, the images captured by HIB project participants in Vietnam were used in a photo-elicitation process, which assisted a large group of farmers, who had not previously been involved in the project, to identify their training priorities. Hence, the long-term legacy of PVM processes needs to be considered, including what sustainability might look like, whether it is feasible and what is required to support it.

In conclusion, our community engagement activities in four very different country contexts have shown the potential of PVM to surface new knowledge that can contribute to public health debates and global health science research. As facilitators of these processes, we need to further explore ways in which researchers, health providers and wider community audiences can be supported to engage in the production and viewing of visual outputs. By doing so we can aim to foster the respect and better understanding needed to build more trusting relationships between researchers and community members and open up opportunities for knowledge exchange, co-learning and fresh perspectives. There are multiple ethical dimensions that need to be considered and acted upon at each step in these visual processes. Although some of the ethical situations that we have encountered in our four settings have previously been described by PVM practitioners working in other fields, there are some which are especially relevant to engagement in global health and health science research that require further study.
